# Nutrition Program Improves Health-Related Outcomes of Non-Diabetic Elderly at Nutritional Risk

**DOI:** 10.1093/geroni/igab046.3715

**Published:** 2021-12-17

**Authors:** Cory Brunton, Luis Carlos Venegas-Sanabria, Gabriel Gomez, Juan Diego Misas, Suela Sulo

**Affiliations:** 1 Abbott Nutrition, Columbus, Ohio, United States; 2 Pontificia Universidad Javeriana, Bogota, Distrito Capital de Bogota, Colombia; 3 Abbott Nutrition, Bogota, Distrito Capital de Bogota, Colombia; 4 Abbott Nutrition, Chicago, Illinois, United States

## Abstract

Malnutrition or its risk affects up to 1 in 3 older adults receiving outpatient care post a hospitalization or for chronic disease management. Although malnutrition poses a negative burden on someone’s recovery and health preservation, it can be effectively addressed through cost-effective nutrition interventions delivered as comprehensive quality improvement programs (QIPs) aiding to advance healthcare professional’s nutrition education/training and improve quality of care for at-risk/malnourished individuals. Although evidence from US and Europe demonstrates nutrition-focused QIPs are effective in delivering high-quality nutrition care and improve health outcomes of outpatients at-risk/malnourished, to date, no evidence has been reported from Latin American countries. We assessed effectiveness of a comprehensive, nutrition-focused QIP in a Colombian outpatient clinic. Between 09/2019-03/2020, 504 (of total 618) QIP participants were classified at-risk/malnourished and non-diabetics. Participants were followed for 90-days either in-person or via telehealth mechanisms (during COVID-19-imposed lockdown period). QIP interventions included healthcare professional nutrition education; QIP participant continuous nutrition and exercise counselling and 60-day supply of oral nutrition supplement (Ensure®, Abbott). QIP participants were 69% female, with >2 comorbidities, and mean age of 73. Improvement or maintenance of good mental health/well-being, frailty status, cognition and quality of life was reported for 90.7% (456/503), 87.3% (407/466), 86.7% (405/467) and 47% (237/504) participants, respectively (p-values<0.05). Results support QIP effectiveness in driving improved health-related outcomes for non-diabetic, at-nutritional-risk participants. These findings highlight the importance of nutrition-focused QIPs with ONS for older adults during their recovery phase post a recent hospitalization and/or for chronic disease management.

